# The role of probiotics on microvascular complications of type-2 diabetes: Nephropathy and retinopathy

**DOI:** 10.34172/jcvtr.32877

**Published:** 2024-06-25

**Authors:** Robab Sarmadi, Hajie Lotfi, Mohammad Amin Hejazi, Fariba Ghiasi, Rana Keyhanmanesh

**Affiliations:** ^1^Stem Cell Research Center, Tabriz University of Medical Sciences, Tabriz, Iran; ^2^Department of Physiology, Faculty of Medicine, Tabriz University of Medical Sciences, Tabriz, Iran; ^3^Cellular and Molecular Research Center, Research Institute for Prevention of Non-Communicable Disease, Qazvin University of Medical Sciences, Qazvin, Iran; ^4^Student Research Committee, Qazvin University of Medical Sciences, Qazvin, Iran; ^5^Food Biotechnology Research Institute, Agricultural Biotechnology Research Institute of Iran, Agricultural Research Education and Extension Organization (AREEO), Tabriz, Iran; ^6^Drug Applied Research Center, Tabriz University of Medical Sciences, Tabriz, Iran

**Keywords:** Probiotics, Gut microbiota, Type 2 diabetes, Retinopathy, Nephropathy, Microvascular

## Abstract

Diabetes is a multifactorial disorder that involves several molecular mechanisms and is still one of the key global health challenges with increasing prevalence and incidence. Gut microbiome dysbiosis could activate and recognize receptors that trigger the inflammation response and modulation of insulin sensitivity. In addition, the intricate role of gut microbiota dysbiosis in the onset and development of T2D (Type 2 diabetes mellitus) and associated microvascular complications was identified. These complications include diabetic nephropathy (DN) and diabetic retinopathy (DR), diabetic neuropathy, cerebrovascular disorders, and coronary heart disease. A recent interesting strategy to improve these complications is probiotics administration. The safety and health effects of probiotics against various diseases have been validated by various in vitro, in vivo and clinical studies. In this review, the related mechanisms between the gut microbiome, initiation, and progression of T2D and its common microvascular complications (DN and DR) have been discussed.

## Introduction

 Diabetes is a chronic and non-communicable disorder with an increasing incidence worldwide that is estimated to reach 783 million cases by 2045.^1^ In recent times, diabetes has been classified into four categories: type 1 diabetes (T1D, destruction of β-cell), type 2 diabetes (T2D, losing of β-cell insulin secretion), gestational diabetes, and diabetes due to alternative factors (monogenic diabetes syndromes, disease related to the exocrine pancreas or induced by drugs/chemicals after HIV/AIDS management by glucocorticoids or following organ transplantation.^2^

 It has been reported that most adults with T2D (98%) suffer from common diabetes-related complications that include overweight and obesity (78.2%), cardiovascular disease (21.6%), hypertension (82.1%), hyperlipidemia (77.2%), chronic kidney disorders (24.1%), neuropathy, and amputation.^3^

 Accumulated evidence suggested the critical functions of gut microbiota in host metabolism as well as the development of obesity and T2D pathogenesis.^4^ The key role of the gut microbiota in mediating the therapeutic benefits of bariatric surgery, food control, and antidiabetic medicines has been identified by results from both clinical and animal investigations.^5^ It has been documented that administration of oral antidiabetic medications such as acarbose and metformin could alter gut microbiome symbiosis and restrict the signaling of gut bile acids which consequently leads to the limited microbial metabolism of bile acids.^6^ Regarding the effect of the gut microbiome in obesity induction, the importance of bile acid signaling in the establishment of bacterial therapeutic effects for T2D is highlighted.^7^ Also, the gut microbiota principally contributed to the digestion of fermented indigestible oligosaccharides and carbohydrates as well as the synthesis of short-chain fatty acids (SCFAs) including acetate, butyrate, and propionate.^8^ The degree of gut microbial dysbiosis in T2D patients is reported to be moderate^9^ and recently the role of gut microbiota in managing diabetes has been presented.^10^

 Probiotics are living microorganisms that provide health-beneficial effects to the host when administered in proper concentrations. Although the effects of the probiotics on T2D are inconsistent, evidence from animal investigations confirmed the efficient role of probiotics in glucose metabolism and increasing cellular sensitivity to insulin.^11^ Administration of probiotics is reported to efficiently decrease fasting blood glucose (FBG), glycosylated hemoglobin (HbA1c), and insulin resistance, which are critical indicators for glucose metabolism deficiencies.^12^

 The abundance of several probiotics including *Lactobacillus spp., Verrucomicrobia phyla*, and *Bifidobacterium spp.,* that pose anti-inflammatory effects are decreased in diabetic patients.^13^ In addition, the abundance of other species bacteria such as *Bacteroides* which can change the gut mucus and alter the glycocalyx barrier is increased in T2D patients. This can be considered a key biomarker for the detection of diabetes in the early status.^14^

**Figure 1:**
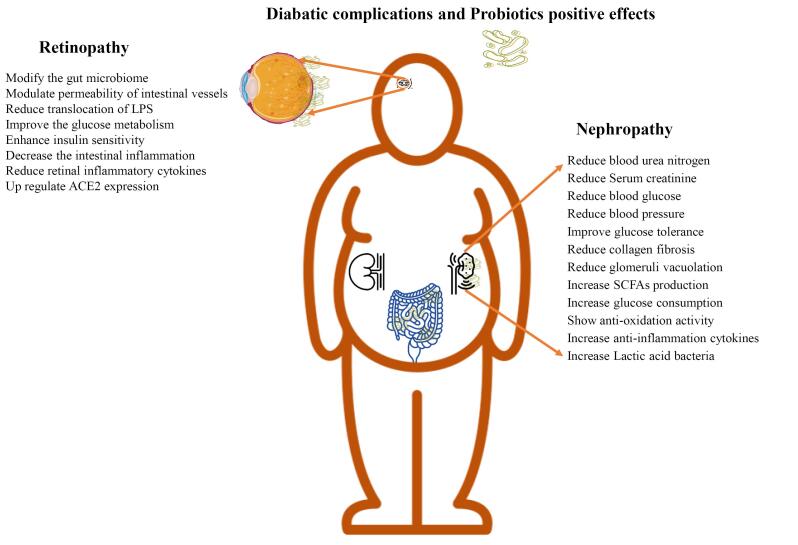
The brief effect of probiotics on diabetic nephropathy and retinopathy

 Due to the impact of probiotics in metabolic disease treatment, in this review, the application of probiotics for T2D treatment and their effects on diabetic-associated microvascular compilations including nephropathy and retinopathy are reviewed which is summarized in Figure 1 as a graphical abstract.

## Probiotics, prebiotics, and synbiotics association with diabetes interventions

 T2D is associated with hyperglycemia, insufficient insulin production, and insulin resistance (IR). The glucose was released inappropriately by the liver into the blood due to inadequate insulin followed by progressive dysfunction of beta cells. Various drugs such as metformin and vildagliptin were prescribed to T2D patients for glycemic control. The related mechanism of drugs refers to raising the utilization and production of insulin, reducing sugar absorption and production, increasing glucose urinal excretion, and stopping the re-absorption of glucose.^15^ However, serious drawbacks are reported after administration of common drugs including allergic reactions, disabled diarrhea, lactic acidosis, arthralgia, hypokalemia, and adverse effects at the injection site due to repeated insulin application.^16^ Although traditional strategies are cost-effective, unsuitable efficacy, cardiovascular complication, and the necessity of long-term administration limit their application.^17^ Therefore, alternative approaches especially based on the human diet that contains probiotics are more interesting. These friendly microorganisms are considered as parts of intestinal microflora, desirable functional foods, and dietary supplements with perceived health effects.^18^

 Probiotics are defined as live organisms, that when administered in adequate amounts, offer health benefits to the host.^19^ Dysbiosis of gut microbiota has been reported in T2D, leading to changes in host obesity, dysfunction of b-cells, metabolic disorders, systemic inflammation, and oxidative stress.^20^ Administration of the probiotics (certain strains of *Lactococcus*, *Lactobacillus*, and *Bifidobacterium*), prebiotics (dietary modification/diets rich in non-digestible fiber), synbiotics (combinations of pre-and probiotics), postbiotics (SCFAs as bacterial fermentation products) and transplantation of fecal microbiota can modulate the gut microbiota.^20^ The probiotics are administered orally and show health benefits. In addition to modulating intestinal microbiota diversity, probiotics can counteract T2D metabolic disorders^21^ by increasing the insulin-sensitizing hormone and reducing liver gluconeogenesis (*L. rhamnosus GG,), *and down regulation of pro-inflammatory genes *(L. curvatus HY7601 *and* L. plantarum KY1032).*^22^ The well-known probiotics effects in the prevention, intervention, and treatment of diabetes have well been reviewed.^23^ The list of probiotics in T2D management is summarized in Table 1.

**Table 1:** Probiotics intervention mechanisms in T2D management

**Probiotics**	**Administrated form **	**Study model**	**Mechanisms**	**Ref**
*L. acidophilus* *L. fermentum* *L. gasseri* *L. rhamnosus*	Live form	Epithelial T84 cell lines	Preserve junction proteins ( E-cadherin and β-catenin)	^24^
*L. reuteri* GMNL-263	Suspension of probiotics (10^9^ CFU/ml) in sterile water	High-fructose fed rats	Unregulated peroxisome proliferator-activated receptor alpha and GLUT4 Down regulated the Srebp-1c, FAS, and Elvol6 as lipogenic genes	^25^
VSL#3	Capsule contain live and freeze dried 450 billion of 8 strain	High fat dieted (HFD)C 57J/B6 male mice	Prevent obesity and diabetes by butyrate and glucagon-like peptide-1 (GLP-1) induction	^26^
*L. plantarum* *L. fermentumL. rhamnosus* (LGG)	viable probiotic (~ 1.5 × 10^9^ colonies)	Diabetic C57BL/6J male mice	Increasing incretins such as GLP-1 and glucose dependent insulin otropic polypeptide	^27^
*L. acidophilus* NCFM	Capsules contain 10^10^ CFU	T2D males	Preserve insulin sensitivity	^28^
*L. acidophilus* *L. casei* *B. bifidum*	2 × 10^9^ CFU/g of probiotics + inulin (800 mg)	Fat diabetic patients which had coronary heart disease	Improve the insulin metabolism and HDL levels	^29^
*B. adolescentis*	Live 5 × 10^8^ CFU/ml	HFD fedrats	Glucose tolerance and insulin sensitivity was improved	^30^
*B. animalis*	Live	HFD fed mice	-Reduce the inflammatory cytokines/responses -Reduce lipopolysaccharides (LPSs)	^31^
*B. longum* *L. rhamnosus* GG (LGG)	2 × 10^9^ of live bacteria	HFD rat	-Up-regulation of RegI protein involved in diabetes	^32^
*L. acidophilus* *S. cerevisiae*	0.25 × 10^11^/mL of Lactobacillus and 0.25 × 10^9^/mL of S. cerevisiae	HFD fed mice	Reduce metabolic and oxidative stress such as alanine aminotransferase, aspartate aminotransferase, glutathione, peroxidase, superoxide dismutase, catalase, malondialdehyde levels	^33^
*B. lactis HY8101*	cell-free extract	L6 cell lines Mice model	Uptake of insulin-stimulated glucose improved via GLUT4 and AKT pathways	^34^
*L. sporogenes*	1 × 10^8^ CFU plusprebiotic (0.07 g inulin )	78 diabetic patients	Decreased serum TG and V-LDL levels Increased HDL	^35^
*L. plantarum* WCFS1	Suspension was prepared by 10^12^ freshly *L. plantarum WCFS1, *saline and glucose	Healthy nonsmoking individulas	Regulate the epithelial integrity by increasing zonulaoccludens	^36^
*L. casei*	10^8^ CFU/day	44 diabetic patients	Reduced serum fetuin-A level, FBS, HOMA.IR index and serum fasting insulin level Increased SIRT1 No change in HbA1c	^12^
*L acidophilus *(2 × 10^9^ CFU)*, L. casei*(7 × 10^9^ CFU), *L. rhamnosus*(1.5 × 10^9^ CFU)*, L. bulgaricus *(2 × 10^8^ CFU), *B. breve *(3 × 10^10^ CFU)*, B. longum* (7 × 10^9^ CFU)*, Streptococcus thermophilus *(1.5 × 10^9^ CFU)	Seven viable and freeze-dried strains	68 diabetic patients	Reduce HDL No significant effect on TC, TG, and anthropometric indexes	^37^

Total cholesterol ( TC), triglyceride (TG), low-density lipoprotein (LDL), and high-density lipoprotein (HDL), Fasting blood sugar (FBS), homeostasis model of assessment-estimated insulin resistance (HOMA-IR).

 In Table 1, various mechanisms that can prohibit the initiation, and development of T2D are explained. Nonetheless, the use of dead or live probiotics is one of the challenges during T2D intervention. It is suggested that dead forms of the probiotics are more desired due to the higher safety and long shelf lifetime.^38^

 Li et.al examined the anti-diabetic impact of multi-strains of five *Lactobacillus* strains (*L. casei*, *L. plantarum*, L. rhamnosus, L. brevis, and *L. plantarum*) in both dead and live forms (contain 8 × 10^10^ CFU/ mL) on diabetic mice models which induced by HFD and streptozotocin. Administration of both live and dead probiotics for 10 weeks was done. Both forms of probiotics decreased leptin and HbA1c levels, and improved glucose tolerance, but the live form of probiotics remarkably reduced the level of fasting and postprandial blood glucose. Also, live probiotics greatly affected insulin resistance and relieved hypoglycemic symptoms significantly. Further, both forms of probiotics could effectively influence the inflammation indicators such as TNF-a, IL-6, and IL-10. Importantly just live form of strains delay the onset of diabetes and recover insulin resistance more efficiently.^39^

 Similar to the previous study, Honda et al assayed the effect of viable and heated cells of *L. rhamnosus* GG and *L. bulgaricus* on normal and KK-A mice (genetic model for T2D) for 6 weeks. The live cells of *L. rhamnosus* can reduce FBG, postprandial blood glucose, and HbA1c at significant levels while these effects were not found after administration of live *L. bulgaricus*. In KK-A mice, after 3 weeks the live *L*. *rhamnosus* could inhibit FBG and improve glucose tolerance significantly, but no positive effect was seen by heat-treated. These outcomes confirmed the potential of *L.rhamnosus* in reducing the postprandial blood glucose in normal mice but the anti-diabetic effect of probiotics on the KK-A mice depends on the live or dead form of the bacterial cell which colonized in the mice gut.^40^ It is concluded that mixed probiotic strains are more effective compared to single-strain, but the dead forms are safer for long-term diabetes intervention, especially when used as food ingredients. However, the answer to the safety aspect remains ambiguous^41^ and further clinical trials are necessary to find the effects, viability, and anti-diabetic activity of probiotics after preparation for schedule prescription.^42^

## Diabetic complications

###  Diabetic nephropathy

 Diabetic nephropathy (DN) is known as a main cause of end-stage renal disease worldwide. The main risk factors for the development of DN are chronic hyperglycemia, dyslipidemia, TC, TG, LDL, and HDL, high blood pressure, and abdominal obesity.^43^ DN formation occurs after different latency years in diabetic patients. Microalbuminuria screening (30-300mg albumin excretion over 24h during 3-6 months in 2 of 3 urine samples) and DN prediction using personal medicine is a major challenge for intensive treatment or preventative approaches.^44^ In T2D, non-proteinuria and non-retinopathy DN are more common due to the activity of renin-angiotensin (RAS) blocker therapy following persistent albuminuria.^45^ Elaborated pathogenesis of DN is associated with poor therapeutic outcomes, in which typical treatment and blood sugar control are not efficient enough to stop the progression and prohibit mortality of DN.^46^ The development of novel treatment approaches depends on an accurate understanding of the pathogenesis of DN. These main pathways and mediators are oxidative stress, angiotensin II (Ang-II), and inflammatory mechanisms.^47^ In the following section, the probiotics potentials in these pathways is discussed as an innovative treatment strategy, especially in the case of inflammation.

## Role of probiotics in controlling diabetic nephropathy

 Based on a plethora of investigations, gut microbiota dysbiosis, and low-grade inflammation are considered highly potential therapeutic agents in the treatment of DN.^48^ The metabolism pathway is a key factor in the illumination of the correlation between gut microbiota and DN.^49^ Dietary polysaccharides are hydrolyzed and fermented by gut microbiota and are subsequently absorbed to use as energy source by the host.^50^ Also, the association of gut-derived endotoxins such as LPS with chronic inflammation, which is a main sign of DN has been documented.^51^

## In vivo studies on probiotics and diabetic nephropathy

 Various studies on DN animals have shown promising outcomes to ameliorate DN symptoms. Kuo et.al investigated the effect of three probiotics (*L.acidophilus*, *B.longum*, and *B.bifidum*) on the DN mice model. The animals received high and low doses of probiotics (5 × 10^9^ CFU/kg/day and 1 × 10^9^ CFU/kg/day, respectively) for 8 weeks. The reduced level of blood urea nitrogen (BUN), serum creatinine, and blood glucose was presented by probiotics. In addition, urine protein was reduced significantly, and the improvement of blood pressure, and glucose tolerance. Both low and high doses reduced collagen fibrosis, and glomeruli vacuolation. Following probiotics treatment the level of acetic acid (as the main component of SCFAs) was raised and glucose consumption, anti-oxidation, and anti-inflammation (IL-10 levels) activity were better than control group. They concluded that combination therapy of probiotics could attenuate renal function deterioration and blood-glucose changes in the animal model.^52^

 Li et.al showed the activity of gut microbiota and production of SCFAs in DN mice models with renal dysfunction. Mice with severe proteinuria had low Firmicutes bacteria. A positive correlation between Allobaculum and body weight and blood glucose level was found. The Anaerosporobacter genus was positively linked with urinary protein content while the Blautia genus had a negative correlation. These results confirmed the role of Allobaculum and Anaerosporobacter in boosting renal dysfunction compared with Blautia in DN mice.^53^ In conclusion, the probiotics potentials to modulate gut microbiota may also help DN patients.

 Manaer et.al isolated four lactobacilli (*L.kefiranofaciens, L.plantarum, L.helveticus, Lactococcuslactis*)and *Issatchenkiaorientalis* then mixed them with camel milk to investigate their effects on metabolisms of lipids and glucose, renal and liver activity, and gut flora in mice models. After 6 weeks of treatment ( with 1.0 × 10^8^ CFU/mL and 1.0 × 10^10^ CFU/mL of probiotics), various analyses showed that these probiotics reduced FBS, oral glucose tolerance test, HbAlc, and IR index, plasma total cholesterol, TG, LDL-C, 24 h urinary microalbumin, urine ketone, and urine sugar at significant levels. Also, C-peptide and HDL-C were increased. The function of the liver and kidney was improved and analysis of gut microbiota presented high frequency of lactic acid bacteria and Bifidobacterium and low Escherichia.^54^

 Another intersected probiotic is *Saccharomyces boulardii* which showed renoprotective potential. Diabetic mice received S.boulardii orally for 2 months. It was found that *S.boulardii* decreased blood glucose but increased the C-peptide significantly. Also, various physiological and biochemical parameters were attenuated after administration. This probiotic can restore the concentration of serotonin and dopamine and raise renal angiotensin (1–7) besides the ACE and ACE2 activities. Renin-angiotensin system and anti-fibrotic axis were activated. It also modulates the intestinal microbiome by reducing the *Proteus*, *Akkermansia*, *Escherichia*, and *Shigella.*^55^

 Tungsanga et.al explored the positive renoprotective effect of *L.rhamnosus* (1 × 10^6^ CFU) on renal injuries by reducing the creatinine, proteinuria, gut-derived uremic toxins, endotoxin, and TNF-α at significant levels on 5/6 nephrectomy mice. At cell-based assays, (Caco-2, HK2 cells), the level of IL-8, NF- κB, types III /IV collagens, TNF-α, and IL-6 were attenuated which presented the potential of probiotics to preserve enterocytes integrity and protect renal fibrogenesis.^56^

 Another investigation by Mihailović confirmed that *L.paraplantarum* can activate responses to protect diabetic-damaged liver and kidney. These activated pathways are related to activation of Akt kinase, reducing DNA damage and procaspase3 degradation along with lowering the inflammatory pathways. Also, this probiotic could attenuate fibrotic stages in the liver and kidney by raising the E-cadherin and decreasing the muscle actin and fibronectin.^57^

 Given that, Feng et al investigated the exact mechanism and correlation between the gut and kidney using two *Bupleurum* polysaccharides from different sources. They established mice models of diabetes using streptozotocin (STZ) and investigated the effects of probiotic *Bupleurum* polysaccharides (60 mg/kg) oral administration for 42 days. The results indicated a significant association between *Bupleurum* polysaccharides administration with ameliorated STZ-induced DN. They reported reduced levels of serum creatinine and glucose, as well as urine albumin following *Bupleurum* polysaccharides treatment. In addition, the expression of inflammation markers was suppressed after *Bupleurum* polysaccharides treatment both in the kidney and colon. A higher diversity of the gut microbiota dysbiosis after treatment was reported which positively improved the gut barrier and protective microbiota. Accordingly, *Bupleurum* polysaccharides were used as a potential therapeutic strategy for DN treatment through modulation of inflammation and gut microbiota.^58^

 In conclusion, the administration of probiotics can be suggested as a promising treatment and controlling DN symptoms. Probiotics modulate glucose and lipid metabolism and produce the SCFAs to donate their antioxidant, and anti-inflammatory properties. Future studies must focus on the gut-kidney axis due to the regulation of the gut microbiome by probiotics to highlight combination probiotic therapy in DN complications.

## Clinical trials on probiotics and diabetic nephropathy

 Besides the positive anti-diabetic effect of probiotics, several clinical trials reported that dietary supplements mixed with probiotics can manage kidney function and DN complications.

 The advantageous impacts of fortified soy milk with *L. plantarum A7* on the biomarkers of kidney function in 48 DN patients were evaluated. Individuals were divided into probiotic-consuminggroup (200 mL/day) and a control group consuming soy milk. Serum levels of cytokine receptor soluble tumor necrosis factor receptor 1 (sTNFR1) which is an inflammatory adipokine progranulin (PGRN), as well as kidney function biomarkers (cystatin C and neutrophil gelatinase-associated lipocalin (NGAL) were analyzed after two months of probiotic consumption. Accordingly, a significant reduction in serum levels of PGRN and cystatin C in the probiotic receiving group compared to the control group was found. While no significant change in the sTNFR1concentration was determined, a marginal significance was observed in the NGAL level in the probiotic*-*consuming group thanthecontrol group.^59^

 Jiang et.al investigated the effect of a probiotic mixture of *B.bifidum* (1.2 × 10^9^ CFU), *L.acidophilus* (4.2 × 10^9^ CFU), *S.thermophiles* (4.3 × 10^9^ CFU) on 76 DN patients with T2D after 12 weeks of probiotic administration. Probiotics could reduce FBS, HbA1c, and microalbuminuria/creatinine levels. In addition, no significant reduction in 2 h postprandial blood glucose and an estimated glomerular filtration rate (eGFR) was observed in patients before and after probiotic consumption.^60^

 AbdelQadir et al reported the beneficial effects of *Bifidobacterium* and *Lactobacillus* in DN patients in a systematic meta-analysis study. The results demonstrated a significant decrease in insulin resistance and HOMA-IR as well as MDA, hs-CRP, and sodium in diabetic patients consuming probiotics supplementation. On the other hand, they resulteda significant increase in TAC, while no significant changes were identified in the lipid profiles as well as biomarkers of oxidative stress and kidney function parameters such as glomerular filtration rate and creatinine in probiotics-consuming diabetic patients. According to their findings, probiotic supplementation could effectively reduce serum levels and IR, and enhance lipid profiles and kidney function, while it has no beneficial effect on kidney function with moderate positive effects on some oxidative stress biomarkers.^61^

 The probiotics’ efficacy in modulating anthropometric indices and lipid profiles in DN patients was determined in a meta-analysis study performed by Moravejolahkami et al according to the results, consumption of the probiotic was effectively correlated with reduced levels of lipid biomarkers (except HDL) while anthropometric indices were non-significantly increased.^62^

 Arani et.al conducted a study to investigate the effects of honey probiotics on metabolic status in 60 DN patients. One group of patients received probiotic honey containing *Bacillus coagulans T11* (10^8^ CFU) and the control group consumed honey for 12 weeks. Then glycemic status, fat concentration, inflammatory biomarkers, and oxidative stress were evaluated after getting a fasting blood sample. Accordingly, serum levels of insulin represented a significant decrease in diabetic patients receiving probiotic honey, as well as assessment-estimated IR and total-/HDL-cholesterol, serum high-sensitivity C-reactive protein (hs-CRP), and plasma malondialdehyde (MDA) levels than the control group. Moreover, a significant increase in the insulin sensitivity check index was observed in diabetic patients consuming probiotic honey. However, other metabolic profiles have no significant different between the two groups.^63^

 According to the analysis of a systematic study, Bohlouli et al demonstrated decreased levels of MDA and hs-CRP in DN patients along with enhanced amounts of total antioxidant capacity (TAC)and glutathione, indicating improved hs-CRP and oxidative stress biomarkers in ND patients receiving probiotic supplements.^64^

 In a recent clinical trial, probiotic effects on the lipid concentrations, glycemic control, plasma levels of oxidative stress, and inflammation biomarkers were evaluated. The study enrolled 60 DN patients undergoing hemodialysis and indicated that probiotic application is effectively linked with reduced serum insulin levels, fasting plasma glucose, HOMA-IR, HbA1c, and homeostasis model of assessment-estimated beta-cell function along with improved insulin sensitivity index. In addition, after 12 weeks of administrating *B.bifidum, L.acidophilus,* and *L.casei*mixtureMDA, hs-CRP, and total iron-binding capacity were significantly reduced in serum levels of the probiotic consuming DN group thanthe control group. Meanwhile, the total level of antioxidant biomarkers was increased by probiotic supplementation in DN patients.^65^

 Likewise, Mafi, et al examined the probiotic effects on the genetic profiles metabolic and metabolism of DN patients as well as lipid profiles, inflammation biomarkers and oxidative stress by a randomized clinical trial along with a HOMA-IR. 60 DN patients were divided into probiotic supplement receiving and placebo-controlled groups. Administration of a probiotic mixture containing 2 × 10^9^ of each *B. bifidum strain ZT-B1, L.acidophilus strain ZT-L1, L.fermentum strain ZT-L3*, and *L.reuteri strain ZT- Lre* for 12 weeks led to significantly decreased fasting plasma glucose and HOMA-IR as well as serum levels of insulin and the quantitative insulin sensitivity check index. Moreover, total-/HDL-cholesterol ratio, and triglycerides, as well as advanced glycation end products, MDA, and hs-CRP, were reduced with probiotic-consumption compared to the placebo intake. Also, a significant raisingin serum levels of HDL-C and glutathione was found in probiotics receiving DN patients compared to the placebo receiving group. The result indicated beneficial effects of probiotic supplements on glycemic control and the beneficial effects on cardio-metabolic risk as well.^66^

 Ultimately, documents indicate beneficial effects of the probiotics as advantageous therapeutic agents in the treatment and controlling DN and associated complications. However, further studies are required to provide detailed insights into the accurate probiotics role supplementation in the expression of inflammatory markers and related mechanisms of DN management.

## Probiotics and diabetic retinopathy

 Diabetic retinopathy is another diabetes complication in which the blood vessels are damaged in the retina due to high blood sugar levels. This damage can gradually lead to vision problems or even blindness. During the early stage of DR, non-proliferative diabetic retinopathy (NPDR), the retinal blood vessels start to leak. However, in the advanced stage of DR, proliferative diabetic retinopathy (PDR), the growing of fragile new blood vessels in the retina and vitreous is observed. Vessel bleeding can cause fluctuating vision, inaccurate color vision, and ultimately vision loss. In addition, this is characterized by several symptoms including hyperpermeability, vasculopathy, neoangiogenesis, and hypoperfusion. The progression of the disease is accompanied by diabetic macular edema, retinal detachment, and vitreous hemorrhage.^67^ According to the global report on diabetes, 1.9% of moderate to severe visual damage was caused by DR in 2010 and it is estimated to reach 700 million cases by 2045.^68^ It has been documented that the functional and morphological manifestations of diabetes initially occur in the neural retina which is associated with angiopathic lesion development.^69^ Various systemic and metabolic disorders including hypertension and hyperglycemia,^70^ diabetes duration,^71^ hyperlipidemia,^72^ and kidney disorders are determined as critical risk factors for DR development according to the epidemiological studies. Current approaches to DR treatment mainly contributed to the alleviation of clinical manifestations in the advanced stages that are related to microvasculature.^73^ There has been a great effort to investigate alternative and comprehensive methods of DR treatment that cover the preclinical symptoms of the disease and improve the glycemic parameters of the patients.^74^

## Influence of gut dysbiosis on the Diabetic retinopathy

 Evidence has demonstrated that deregulation of the microbiota could result in low-grade inflammation both local and systemic through the gut retina axis. This could directly influence DM development and related microvascular impairments including DR.^75^ On the other hand, the correlation between gut microbiota such as phylum Bacteroidetes/Firmicutes with the development of T2D has been reported. The process is associated with enhanced permeability of intestinal vessels and translocation of LPSs.^76^ Gut dysbiosis has been reported to increase the permeability of intestinal vessels which in turn enhances bacterial infiltration and triggers various responses. It has been demonstrated that some gut bacteria influence the initiation of DM while some are capable of stimulating anti-inflammatory responses that help the protection against DM development.^77^ The bacteria *Bacteroidesfragilis, Roseburiaintestinalis, Akkermansiamuciniphila*, *L. fermentum, L. plantarum, L. casei* have been reported to improve the metabolism of glucose by inducing the expression of IL-10. The *R. intestinalis* enhances insulin sensitivity after up regulation of IL-22 which ultimately decreases intestinal inflammation.^77^ Beli et al examined the changes in gut microbiome after intermittent fasting (IF) in DN db/db mice. They found no change in HbA1c but animal survival was longer significantly. Acellular capillaries and leukocyte infiltration were reduced. Their focus was on the changes in the gut microbiome and the production of beneficial metabolites to inhibit DR development. The results presented high Firmicutes and low Bacteroidetes in IF mice which led to rising gut mucin, villi length, and reduction of plasma peptidoglycan. On the other hand, tauroursodeoxycholate (TUDCA) level was increased which is a neuroprotective bile acid due to the association of Firmicutes on metabolisms of bile acid. Conclusively, IF can improve the species to produce TUDCA and retinal production by activation of TGR5 (as TUDCA receptor in the retinal primary ganglion cells).^78^ Accordingly, DR was characterized by changes in specific operational taxonomic units (OTU) that are mainly attributed to the families *Atopobiaceae*, *Acidaminococcaceae*, *norank_o_Coriobacteriales*, and *Muribaculacea*. The twenty-two genera referred to DR as well as eight genera characterizing diabetes without retinopathy and thirteen genera for the healthy group were found. It is suggested that the microbiota of DR patients might tend to demonstrate complex pathological diversity. The variable populations of *Bacteroidetes*, *Firmicutes*, and *Desulfobacterota* phyla was reported to be associated with the development of DR. Moreover, high levels of *Bifidobacterium*, *Lactobacillus*, and *Blautia* along with low levels of *Clostridium*, *Faecalibacterium*, and *Eubacteriumhallii* group were reported to be attributed to both DR and diabetes without retinopathy.^79^ In a recent study, Floyd and Grant explained the gut–eye axis associations with potential therapeutic approaches including fecal microbial transplant, intermittent fasting, and consumption of pre-and probiotics and antibiotics. According to the report, gut microbial dysbiosis could induce immunologic responses such as inflammation that result in the local or distal destruction of retina tissue.^80^

## Effects of probiotics on the in vivo models of diabetic retinopathy

 Using probiotic species *L. paracasei*(1 × 10^9^ CFU*, LP)*, Verma et al designed a recombinant system as a live delivery vector, expression, and secretion of human codon-optimized angiotensin-converting enzyme 2 (ACE2). According to this study, higher ACE2 activity in both serum and retina tissue was observed in the mice treated with *LP* fused with the non-toxic cholera toxin. Administration of the *LP* vector decreased the expression of retinal inflammatory cytokines as well as acellular capillaries and successfully prohibited the loss of ganglion cells.^81^ Prasad et al used Akita mice to show the prevention effect of *L. paracasei* (1 × 10^10^ CFU, expressed humanized ACE2) against DR. Overexpression of ACE2 can preserve intestinal barrier integrity, decrease the inflammatory response and delay DR development via lowering the in glucose transporter signaling pathways not by controlling the glucose homeostasis.^82^

 Another recent study has shown a reduction in the disease severity in a mice model of DR following altered feeding patterns that resulted in gut microbiome restructuring. Beli et al identified *Actinobacteria, Bacteroidetes, Firmicutes, Proteobacteria, Tenericutes,* and *Verrucomicrobia*, were the major resident phyla in the fecal microbiome of the DR mice. In addition, no significant increase in *Bacteroidetes *to* Firmicutes *ratio was observed in DR mice compared to the control group.^78^ Interestingly, only 4% of the bacterial microbiome posse considerable identity between the man and mice model of DR.^83^ Several gut microbiota such as *Escherichia coli, Bacteroidetes thetaiotaomicron, *and *Akkermansia muciniphila*, can enhance the permeability of gut and endotoxemia that could mediate chronic inflammatory responses which are of great importance in the DR initiation. ^76^

## Clinical trial of probiotics on the diabetic retinopathy

 In a recent study, Das et al presented different microbiomes relative to the phyla and several other genera in DR patients in comparison with to the T2D patients and healthy control group. According to this study, four phyla including *Proteobacteria, Firmicutes, Actinobacteria, *and *Bacteroidetes *were the most dominant microbiome in T2D patients compared to the control group. Likewise, *Eggerthellalenta*and *Eubacterium* genera were significantly different in T2D and the control group. On the other hand, *Anaerostipes*and*Turicibacter, Blautia, Comamonas, Coprococcus, Haemophilus, Lachnospira, Phascolarctobacterium, Roseburia*, and *Sutterella* were significantly decreased in T2D compared to the control group. Furthermore, *Blautia*and*Anaerostipes, Coprococcus, Lachnospira, Phascolarctobacterium, Roseburia*, and anti-inflammatory bacteria, as well as pro-inflammatory bacteria including *Enterobacter, Escherichia*, and *Methanobrevibacter*and* Treponema,* were lower in T2D compared to the control group. Anti-inflammatory genera including *Bifidobacterium, Butyrivibrio, Faecalibacterium, Lactobacillus, Mitsuokella, Ruminococcus*, and *Streptococcus*, as well as pro-inflammatory bacterium *Sutterella*, probiotic bacterium *Lactobacillus*, and several pathogenic bacteria (*Bulleida, Comamonas, Clostridium, Desulfovibrio, Erwinia, Haemophilus, *and *Rothia*), were considerably lower compared to the T2D and control group.^84^ Altogether, the modified balance in the gut microbiome, as well as the presence of pathogenic organisms, are key factors that could influence DR status. However, there is still a necessity for further detailed investigations to ensure the direct impact of gut dysbiosis in retinopathy in the diabetic patients. These promising results present the feasibility and efficacy of probiotics to modify the gut microbiome and engineered species to activate the main pathways to prevent DR development.

## Discussion

 The significant beneficial effects of probiotics on DR and DN are modulating glucose and lipid metabolism, producing SCFAs, and enhancing insulin sensitivity with antioxidant and anti-inflammatory potential. In the context of DN, probiotics play a role in addressing chronic microvascular disorders characterized by hyperpermeability, vasculopathy, neoangiogenesis, and hypoperfusion. Additionally, probiotics can reduce blood urea nitrogen, serum creatinine, blood glucose, blood pressure, collagen fibrosis, glomeruli vacuolation, and improve glucose tolerance.^60,63,85-87^ As a final discussion, to improve the dysbiosis of gut microbiota, renal metabolic factors, and the reduction of DN progression, administering single or combination of multi-species probiotics within proper dosage and duration can be the main nutritional treatment.^88^ Wang et al reported a negative relationship between foods with high microbial groups (including probiotics) and the development of diabetic kidney complications among 1467 participants. Individuals consuming high-live microbe foods had low HbA1c and serum creatinine levels, confirming the beneficial impact of probiotics on slowing the advancement of kidney disease in diabetics.^89^ In addition, Jin et.al identified the causal relationships between 11 microbial species in DN patients and suggested that probiotics (SCFAs-producing or LPS-inhibiting species) can improve the gut-kidney axis as new nutritional strategies.^90^ Furthermore, probiotics can potentially suppress the expression of inflammation markers in both the kidney and colon, positively improving the gut barrier and protective microbiota. The beneficial effects of probiotics especially, *Bifidobacterium* and *Lactobacillus* species, open new avenues to support intestinal barrier integrity and immunomodulatory consequences because the balancing of Firmicutes/Bacteroidetes ratio through probiotics can reduce systemic inflammation, enhance insulin sensitivity, and mitigate retinal damage in T2DM.^91^ Probiotics could mitigate the DR progression, including diabetic macular edema, retinal detachment, and vitreous hemorrhage. In addition, probiotics can modify the gut-retina axis,^92,93^ modulate the permeability of intestinal vessels, reduce translocation of LPSs, reduce fasting glucose (*Streptococcus salivarius*),^94^ enhance insulin sensitivity,^95^ decrease intestinal inflammation and retinal inflammatory cytokines, and finally up-regulated the ACE2 expression.^52,96^ Also, probiotics can delay DR progression by reducing retinal peripapillary /damaging of endothelial cells and improving the dysfunction of retinal microcirculatory.^97^ These findings suggest that probiotics could offer a promising treatment and control of diabetic retinopathy and nephropathy, potentially providing a novel addition to pharmaceutical treatments for these conditions.

## Conclusion

 Recent advances in methodological approaches for genome sequencing of bacteria have accelerated an accurate understanding of the correlation between gut microbiomedysbiosis association and the development of several pathologies including diabetes. Significant progress has been established toward the identification of the elaborated chemical interactions between gut dysbiosis and host health, especially at the cellular and molecular levels. However, determining the gut microbiome roles in the prevention, management, and treatment of diabetes as well as associated microvascular and macrovascular complications still requires further investigations in detail. The state of gut dysbiosis is linked with the diabetes developments and microvascular complications such as DN and DR. As mentioned throughout our review, diabetic complications are mostly characterized by systemic inflammation and dysfunctions of several organs such as the gastrointestinal, coronary system, kidneys, and eyes that are mainly resulted from ling-term hyperglycemia. Therefore, the majority of investigations have focused on the illumination of probiotics’ certain role in inflammation and determining their effects on the functional biomarkers as well as modulating insulin serum levels and resistance. During the past decades, several animals and clinical experiments on transplanting gut microbiota indicated the efficient role of probiotics in the cause and the consequences of DN and DR. Reducing probiotics population with anti-inflammatory properties along with high frequency of pro-inflammatory probiotics are reported to be attributed to the initiation and development of DN and DR. Abnormal metabolite productions by gut microbiome including SCFAs and LPs are associated with pathogenesis of T2D and related microvascular complications through immunologic and metabolic pathways. Previous literatures suggested that the growth of pre- and probiotics have a positive effect in the protection against T2D development. Ultimately, the application of probiotics could offer a novel addition to pharmaceutical treatments for diabetes. There is an intense interest in developing novel therapeutic dietary agents using beneficial probiotics.

## Acknowledgements

 Authors thanks Drug Applied Research Center of Tabriz University of Medical Sciences.

## Competing Interests

 None to be declared.

## Ethical Approval

 This study was approved by Research Ethics Committees of Tabriz University of Medical Sciences. Ethical Code: IR.TBZMED. REC.1400.350.
